# The genetic basis of early-onset hereditary ataxia in Iran: results of a national registry of a heterogeneous population

**DOI:** 10.1186/s40246-024-00598-5

**Published:** 2024-04-03

**Authors:** Nejat Mahdieh, Morteza Heidari, Zahra Rezaei, Ali Reza Tavasoli, Sareh Hosseinpour, Maryam Rasulinejad, Ali Zare Dehnavi, Masoud Ghahvechi Akbari, Reza Shervin Badv, Elahe Vafaei, Ali Mohebbi, Pouria Mohammadi, Seyyed Mohammad Mahdi Hosseiny, Reza Azizimalamiri, Ali Nikkhah, Elham Pourbakhtyaran, Mohammad Rohani, Narges Khanbanha, Sedigheh Nikbakht, Mojtaba Movahedinia, Parviz Karimi, Homa Ghabeli, Seyed Ahmad Hosseini, Fatemeh Sadat Rashidi, Masoud Garshasbi, Morteza Rezvani Kashani, Noor M. Ghiasvand, Stephan Zuchner, Matthis Synofzik, Mahmoud Reza Ashrafi

**Affiliations:** 1 Pediatric Neurology Division, Pediatrics Center of Excellence, Ataxia Clinic, Children’s Medical Center, Tehran University of Medical Sciences, Tehran, Iran; 2 Cardiogenetic Research Center, Rajaie Cardiovascular Medical and Research Institute, Iran University of Medical Sciences, Tehran, Iran; 3 Pediatric Headache Program, Barrow Neurological Institute, Phoenix Children’s Hospital, Phoenix, AZ USA; 4 Department of Pediatrics, Division of Paediatric Neurology, Vali-E-Asr Hospital, Imam Khomeini Hospital Complex, Tehran University of Medical Sciences, Tehran, Iran; 5 Physical Medicine and Rehabilitation Department, Children’s Medical Center, Tehran University of Medical Sciences, Tehran, Iran; 6 Division of Pediatric Neurology, Department of Pediatrics, Golestan Medical, Educational and Research Center, Ahvaz Jundishapour University of Medical Sciences, Ahvaz, Iran; 7 Department of Pediatrics, Division of Paediatric Neurology, Mofid Children’s Hospital, Shahid Beheshti University of Medical Sciences, Tehran, Iran; 8 Department of Neurology, School of Medicine, Hazrat Rasool-E Akram General Hospital, Iran University of Medical Sciences, Tehran, Iran; 9 Children Growth Disorders Research Center, Department of Pediatric, Shahid Sadoughi University of Medical Sciences, Yazd, Iran; 10 Department of Pediatric Diseases, Faculty of Medicine, Ilam University of Medical Sciences, Ilam, Iran; 11 Department of Pediatrics, School of Medicine, Kerman University of Medical Sciences, Kerman, Iran; 12 Department of Pediatrics, Taleghani Children’s Hospital, Golestan University of Medical Sciences, Gorgan, Iran; 13 Neuroscience Research Center, Shahid Beheshti University of Medical Science, Tehran, Iran; 14 Department of Medical Genetics, Faculty of Medical Sciences, Tarbiat Modares University, Tehran, Iran; 15 Bahrami Hospital, Tehran University of Medical Sciences, Tehran, Iran; 16 Department of Biology, Grand Valley State University, Allendale, MI 49401 USA; 17 Department of Human Genetics and John P. Hussman Institute for Human Genomics, Dr. John T. Macdonald Foundation, University of Miami Miller School of Medicine, Miami, FL USA; 18 Department of Neurodegenerative Diseases, Hertie-Institute for Clinical Brain Research and Center of Neurology, University of Tübingen, Tübingen, Germany; 19 Center for Neurodegenerative Diseases (DZNE), Tübingen, Germany; 20 Department of Pediatrics, Division of Paediatric Neurology, Growth and Development Research Center, Pediatrics Center of Excellence, Children’s Medical Center, Tehran University of Medical Sciences, Tehran, Iran

**Keywords:** Autosomal recessive cerebellar ataxia, Ataxia, Hereditary cerebellar ataxia, Spinocerebellar ataxia, Iranian population

## Abstract

**Background:**

To investigate the genetics of early-onset progressive cerebellar ataxia in Iran, we conducted a study at the Children’s Medical Center (CMC), the primary referral center for pediatric disorders in the country, over a three-year period from 2019 to 2022. In this report, we provide the initial findings from the national registry.

**Methods:**

We selected all early-onset patients with an autosomal recessive mode of inheritance to assess their phenotype, paraclinical tests, and genotypes. The clinical data encompassed clinical features, the Scale for the Assessment and Rating of Ataxia (SARA) scores, Magnetic Resonance Imaging (MRI) results, Electrodiagnostic exams (EDX), and biomarker features. Our genetic investigations included single-gene testing, Whole Exome Sequencing (WES), and Whole Genome Sequencing (WGS).

**Results:**

Our study enrolled 162 patients from various geographic regions of our country. Among our subpopulations, we identified known and novel pathogenic variants in 42 genes in 97 families. The overall genetic diagnostic rate was 59.9%. Notably, we observed *PLA2G6*, *ATM*, *SACS*, and *SCA* variants in 19, 14, 12, and 10 families, respectively. Remarkably, more than 59% of the cases were attributed to pathogenic variants in these genes.

**Conclusions:**

Iran, being at the crossroad of the Middle East, exhibits a highly diverse genetic etiology for autosomal recessive hereditary ataxia. In light of this heterogeneity, the development of preventive strategies and targeted molecular therapeutics becomes crucial. A national guideline for the diagnosis and management of patients with these conditions could significantly aid in advancing healthcare approaches and improving patient outcomes.

## Introduction

The hereditary cerebellar ataxias encompass a group of disorders that exhibit clinical and genetic heterogeneity. These conditions can manifest through various modes of inheritance, with autosomal recessive cerebellar ataxia (ARCA) representing the most complex form. ARCA is characterized by progressive gait incoordination, poor coordination of hands, speech, and eye movements [1–3]. The prevalence of different types of ARCA varies across ethnicities, ranging from 0.0 to 7.2 per 100,000 individuals [4]. More than 100 genes have been identified as causative factors for ARCA [2, 5, 6]. The disorders may present with additional signs such as spasticity, neuropathy, abnormal eye movements, dystonia, and intellectual deficits. Extra-central nervous system signs and symptoms can also be observed in autosomal recessive ataxias, which may aid physicians in pursuing genetic-guided testing during the diagnostic process [1, 7].

Friedreich ataxia (FA), ataxia telangiectasia (AT), ataxia with oculomotor apraxia (AOA), and autosomal recessive spastic ataxia of Charlevoix-Saguenay (ARSACS) are among the most common forms of ARCA. These conditions typically present with a complicated phenotype [4, 7–9]. For instance, FA, the most prevalent recessive ataxia affecting 1 in 50,000 individuals in white populations, is caused by homozygous expansions of an intronic GAA trinucleotide repeat in the *FXN* gene [10, 11]. Some types of ARCAs can be diagnosed based on laboratory findings; for example, ataxia with vitamin E deficiency caused by mutations in the *TTPA* gene, which is common among Mediterranean populations, exhibits a clinical phenotype similar to FA, featuring head tremor, cervical dystonia, and extrapyramidal symptoms [12].

Identifying the underlying genes and related molecular pathways involved in the affected spinocerebellar tracts is a crucial step in understanding the mechanisms of neurodegeneration occurring in cerebellar ataxias. This knowledge can pave the way for the development of targeted treatment strategies. Several studies have shed light on the genetics of some forms of ARCAs [13–15]. Recently, we conducted a review focused on early-onset cerebellar ataxias to establish a practical guideline and outline the most common disorders presenting with early-onset manifestations [submitted]. However, there is limited data on the frequency of these disorders in Iran, which serves as a significant crossroads of the Middle East. In 2018, the Iranian Registry of ARCAs was established by the Tehran University of Medical Sciences with the aim of describing the key demographic, clinical, and genetic characteristics of registered patients. Furthermore, the registry aims to provide genuine access to the worldwide web-based ARCA registry [6].

In this study, we present the genetic basis of autosomal recessive hereditary ataxia in Iran and the results obtained from the national registry, which includes a heterogeneous population. Our findings offer valuable insights and clues to identify the genes and related proteins involved in the pathways within the affected spinocerebellar tracts within this population.

## Materials and methods

### Study design

The study was conducted on patients with early-onset hereditary ataxias at the Children’s Medical Center (CMC), a tertiary referral children’s hospital in Tehran, Iran over a period of 33 months (2019–2022). The research was part of an international collaboration with the Ataxia Research Group at the Hertie Institute for Clinical Brain Research in Tubingen, Germany, and the GENESIS (GEM.app) platform at the Dr. John T. Macdonald Foundation Department of Human Genetics and John P. Hussman Institute for Human Genomics, University of Miami Miller School of Medicine, Miami, FL, United States.

The study received approval from the ethics committee of the Children’s Medical Center and the National Institute for Medical Research Development (NIMAD) of Iran. It was conducted following the ethical standards outlined in the 1964 Declaration of Helsinki and its subsequent amendments.

### Clinical evaluations

In this study, all patients presenting with progressive ataxia before the age of 20 were included, and they underwent a diagnostic assessment and clinical follow-up for a period of two years. The primary focus of the investigation was on autosomal recessive early-onset ataxias (EOAs) with the aim of identifying potential novel genes and variants. Pedigrees exhibiting a probable autosomal recessive mode of inheritance were selected for further study. The patients underwent comprehensive clinical and paraclinical evaluations, and genetic investigations were carried out to better understand the underlying causes of their condition. In order to exclude patients with acquired ataxia resulting from conditions such as infarct, traumatic brain injury, brain infection, and others, brain imaging and medical history were carefully assessed. Subsequently, patients diagnosed with early onset autosomal recessive ataxia were identified and listed in Tables 1 and 2. Furthermore, we specifically considered family pedigrees demonstrating an autosomal recessive mode of inheritance, particularly those with consanguineous marriages.

In this study, the patients were assessed using a Case Report Form (CRF) prepared by the Ataxia Global Initiative (AGI) [16] and the GENESIS platform group, as recommended by international collaborators. Information, data, and the results of clinical evaluations were collected using a designed questionnaire. The severity of ataxia during the clinical course was evaluated using the Scale for the Assessment and Rating of Ataxia (SARA) − 5th version [17]. Furthermore, the presence and severity of non-ataxia signs were assessed using the Inventory of Non-Ataxia Symptoms (INAS) − 6th version, which consists of two parts: one focusing on clinical findings (reflexes, motor symptoms, sensory symptoms, ophthalmological findings), and the other on reported abnormalities (such as double vision, dysphagia, etc.) [18].

Detailed patient histories, clinical evaluations, and relevant investigations, including Magnetic Resonance Imaging (MRI), electrodiagnostic exams (EDX), and various biomarker assessments, such as serum electrolytes, complete blood count (CBC), alpha-fetoprotein, albumin, vitamin E, triglycerides, lipid profile (cholesterol, HDL, LDL), and immunoglobulins (IgG-IgM-IgE), as well as liver, kidney, and thyroid function tests, were thoroughly documented as part of the evaluation process.

In this study, acquired ataxias, including post-infectious cerebellitis, tumors, and congenital structural cerebellar abnormalities, were excluded from the patient cohort. Additionally, patients with autosomal dominant cerebellar ataxias were excluded from the study. Furthermore, to gain a comprehensive understanding of the patients’ conditions, additional clinical evaluations such as audiometry, ophthalmoscopy, urologic, orthopedic, or cardiology consultations were conducted based on their clinical presentations and neurological examination findings. These additional evaluations aimed to gather a broader spectrum of information to aid in the diagnosis and management of the patients with early-onset hereditary ataxias.

### Genetic investigations

#### WES, WGS and mtDNA sequencing

In this study, whole blood samples were collected from both patients and their family members. Genomic DNA was extracted from the samples using standard protocols. To identify causal variants in exonic regions and exon/intron boundaries, Whole Exome Sequencing (WES) was performed. The WES procedure was conducted on the NovaSeq 6000 platform, utilizing the Agilent SureSelect Human All Exon V7 Kit, with an average read depth of 100X.

An in-house bioinformatics pipeline, previously described elsewhere [14], was employed to analyze the WES data. The pipeline consisted of several steps: first, FastQC tool (version 0.11.9) was used to perform quality control of the reads [19]. Next, Bowtie2 (Version 2.4.0) aligning tool was utilized for alignment to the human reference genome (GRCh38/hg38) [20]. The SAM files were converted to BAM (Binary Alignment Map) format using Picard [21]. Local realignment of insertion/deletion (indels) was carried out by employing the Genome Analysis Toolkit Haplotypecaller (GATK) [22]. Variants were annotated using the Ensemble VEP tool and wANNOVAR (http://wannovar.wglab.org/) [23].

To identify potential causal variants, variants with a minor allele frequency (MAF) higher than 1% were removed based on data from the gnomAD database and the 1000 Genomes project. Additionally, the variants were compared and analyzed against various databases, including the Exome Aggregation Database (http://gnomad.broadinstitute.org), Exome Sequencing Project 6500 (http://evs.gs.washington.edu/EVS/), the Exome Aggregation Consortium database (http://exac.broadinstitute.org), and the Greater Middle East Variome Project (http://igm.ucsd.edu/gme/).

For variant analysis, filter settings were applied as recommended by the NGS Ataxia Working Group of the Ataxia Global Initiative [24]. These comprehensive analyses were conducted to identify potential disease-causing variants in the patients with early-onset hereditary ataxias.

#### Repeat expansion analysis

For the molecular diagnosis of FA, GAA repeat expansion analysis was performed as a routine procedure. Briefly, the target region containing the GAA trinucleotide repeats was amplified using triplet repeat primed PCR (TP-PCR), following the methodology described elsewhere [25].

To size fractionate the PCR products, capillary electrophoresis was employed, and this step was carried out using an ABI 3500 Genetic Analyzer. The analysis of the GAA repeat expansion is essential for identifying the characteristic repeat expansions associated with Friedreich ataxia, aiding in the accurate diagnosis of this condition.

#### *In Silico* Analysis

In this study, the ACMG 2015 classification system was utilized to classify the variants. To predict the pathogenicity of these variants, a combination of bioinformatics software tools was applied. The tools used for pathogenicity prediction included:

1. MutationTaster (www.mutationtaster.org/).
2. SIFT (https://sift.bii.a-star.edu.sg).
3. PROVEAN (http://provean.jcvi.org/index.php).
4. CADD (https://cadd.gs.washington.edu/home).
5. HOPE (https://www3.cmbi.umcn.nl/hope/input/).

These bioinformatics tools play a crucial role in assessing the potential impact of genetic variants on protein function and, consequently, their association with disease pathogenicity. The combined analysis from these tools aids in identifying potentially pathogenic variants in the patients with early-onset hereditary ataxias.

##### Confirmation of the variants

To confirm the presence of novel variants and perform segregation analysis, specific primers were designed to target the regions of interest. These regions were then amplified using polymerase chain reaction (PCR). The amplified DNA fragments were subsequently subjected to Sanger sequencing using the Applied Biosystems 3500XL PE Genetic Analyzer.

During the segregation analysis, the identified novel variants were checked among the family members of each family. This step helps to determine whether the variant co-segregates with the disease phenotype within the affected families. By analyzing the variants in the affected individuals and their family members, researchers can establish a clearer understanding of the variant’s role in the hereditary ataxia and its association with the disease.

## Results

### Distribution of ataxias and variants among Iranian subpopulations

A total of 162 patients with early onset ataxia were enrolled in the study during the period of 2019–2022. After exclusion of acquired and autosomal dominant cerebellar ataxias we identified known and novel pathogenic variants in 42 genes in 97 families among our subpopulations. In our study, a total of 88 variants were detected in 42 genes among the patients. These variants comprised 41 missense variants, 40 chain-termination variants (14 frameshift and 25 nonsense variants), and 8 splice variants. The majority of the identified variants were found in *PLA2G6*, with 12 out of 17 variants being missense, followed by *ATM*, where 8 out of 12 variants were nonsense, and *SACS*, where 6 out of 12 variants were nonsense.

Among the detected variants, 45 were novel variants, suggesting potential new genetic causes of the disease. Based on the ACMG 2015 guidelines, these variants were classified as follows: 15 variants were classified as pathogenic, 10 as likely pathogenic, and 20 as Variants of Uncertain Significance (VUS) (see Table 2 for details).

Interestingly, the variant c.272 A > T in the *NPC2* gene was observed in two unrelated individuals, indicating its possible involvement in the disease in these cases. Overall, our findings shed light on a diverse array of genetic variants that may contribute to early-onset hereditary ataxias, and the classification of these variants according to the ACMG guidelines provides valuable insights into their potential impact on disease causation.

Iran’s geographical location at the crossroads of the Middle East has endowed it with significant geopolitical, economic, and cultural influence in the region. Positioned along the ancient Silk Road, Iran has historically served as a bridge or meeting point between Eastern and Western civilizations. This strategic location has contributed to the country’s pivotal role in trade, cultural exchange, and the movement of ideas. Iran’s rich history is marked by numerous ethnicities with distinct cultures coexisting within its borders. The country has been shaped by various historical events, including invasions, wars, and interactions with neighboring countries. The resulting population mixing has led to a diverse genetic composition, especially in regions near Iran’s borders, where the populations can be representative of their neighboring countries.

**Fig. 1:**
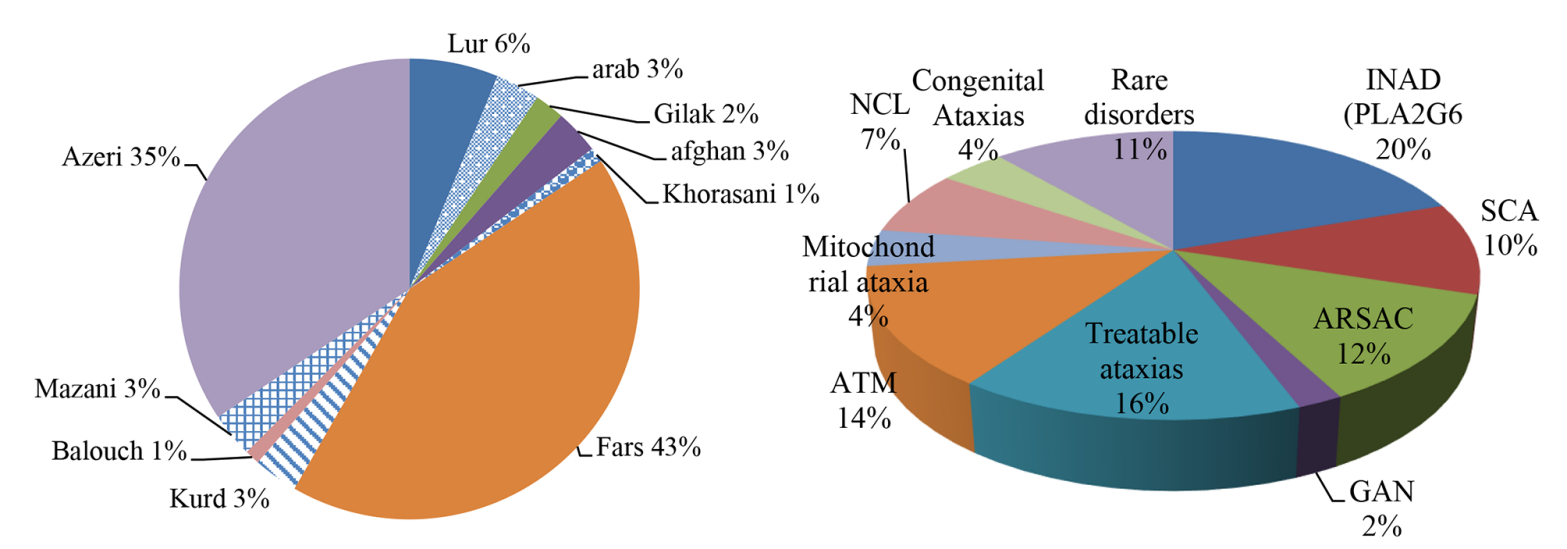
**(A)** Ethnicities of patients; Fars and Azeri were the main cohorts of our study. **(B)** Frequencies of ataxias in this study

In the context of the study, two prevalent ethnicities stood out: Fars and Azeri families. These ethnic groups accounted for 43% and 35% of the cases, respectively (Fig. 1A). The prominence of these ethnicities in the study population highlights the importance of considering the genetic diversity present in Iran, as it may provide valuable insights into the genetic basis of early-onset hereditary ataxias within the broader Middle Eastern region.

**Table 1:** Distribution of common types of ataxia according to the patients’ ethnicities in this study

Ethnicity	INAD	SCA	ARSAC	GAN	Treatable ataxias	ATM	Mitochondrial ataxia	NCL	Congenital Ataxias	Rare disorders	Total
Fars	10	6	3	2	5	6	1	2	2	4	41
Azeri	3	2	6	0	6	4	3	3	1	5	33
Lur	3	0	1	0	1	1	0	0	0	0	6
Arab	1	0	1	0	1	0	0	1	0	1	5
Afghan	1	0	0	0	0	1	1	0	0	0	3
Kurd	0	0	0	0	1	0	0	1	0	0	2
Mazani	1	0	0	0	1	1	0	0	0	0	3
Gilak	0	0	1	0	1	0	0	0	0	0	2
Khorasani	0	0	0	0	0	1	0	0	0	0	1
Balouch	0	0	0	0	0	0	0	0	0	1	1
Total	19	8	12	2	16	14	5	7	3	11	97

### The most common types of ARCAs in Iran

#### *PLA2G6* (INAD)

In our study, all patients except one were found to be homozygous for the identified variants. About 20% of the stuided patients were INAD (Fig. 1B). Among the INAD patients, we detected 12 missense variants, followed by 2 nonsense variants, 2 splice variants, and 1 frameshift variant. The ethnicities of these patients were as follows: 10 patients were of Fars ethnicity, 3 were Lur, 3 were Azeri, 1 was Arab, 1 was Mazani, and 1 was of Afghan ethnicity (Table 1). These findings provide valuable insights into the genetic makeup and distribution of variants among different ethnic groups in our study cohort, which can be crucial for understanding the genetic basis of early-onset hereditary ataxias in diverse populations.

#### Treatable ataxias: 6 novel variants

In our study, we identified five patients with homozygous expansions of GAA repeats, with more than 175 repeats within intron 1 of the *FXN* gene. This expansion is associated with Friedreich’s ataxia (FRDA), an autosomal recessive degenerative disorder caused by dynamic mutations (GAA triplet repeat expansion) of the frataxin gene. These patients with FRDA were treated with the now FDA-approved drug Omexavalone. Most of the patients with FRDA from our study cohort were from the west of Iran (as indicated in Table 1).

In our study, we investigated Ataxia with Oculomotor Apraxia Type 1, which is caused by pathogenic variants in the *APTX* gene. Six individuals from five families were found to have three novel variants in the *APTX* gene which have not been previously reported in the scientific literature (Table 2).

In our study, we investigated different types of hereditary ataxias, including Coenzyme Q10 (CoQ10) deficiency, Ataxia with Oculomotor Apraxia (AOA2), Ataxia with Vitamin E Deficiency (AVED), and Niemann–Pick disease (NPC). Coenzyme Q10 deficiency can result from pathogenic variants in the *COQ2*, *COQ4*, *COQ6*, *COQ8A*, or *COQ8B* gene. Among the patients, we found three individuals with variants in the *COQ8A* gene, and one of them had a novel variant (c.1162G > A). AVED is an autosomal recessive disease caused by pathogenic variants in the *TTPA* gene located on chromosome 8q13. In our study, we identified a novel likely pathogenic variant (c.798del leading to p.Glu267LysfsTer27) in two affected children from one Azeri family. Both patients with AVED presented with dystonia, but no other remarkable clinical symptoms were found. Brain imaging of both cases was normal, and they were placed on vitamin E therapy, although no significant change in their condition was detected during follow-up visits.

Niemann–Pick disease (NPC) is typically due to biallelic pathogenic variants in the *NPC1* or *NPC2* gene located on 18q11, with their encoded proteins having roles in the movement of lipids within cells. In our study, two patients showed variants in the *NPC2* gene. These patients had a novel missense variant (c.272 A > T, p.Asp91Val).

In the subcategory of treatable ataxias, we had 17 patients from 16 families, which included 5 cases of Friedrich’s ataxia, 4 cases of Ataxia with Oculomotor Apraxia, 3 cases of CoQ10 deficiencies, 1 case of AVED, and 2 cases of NPC. All of these patients received appropriate therapy based on their diagnosis. Specifically, all patients were placed on CoQ10 therapy, and 3 of them showed improvement in their condition. These findings highlight the importance of genetic diagnosis and tailored treatments for various forms of hereditary ataxias, particularly in cases where specific therapies, such as CoQ10 supplementation, can lead to positive outcomes and potentially ameliorate the symptoms of the disorder.

#### Ataxia telangiectasia (AT): four novel variants: four novel variants

In our study, we identified several variants in the patients, as shown in Tables 2 and 3. Among the patients, 15 individuals from 14 unrelated families were found to have pathogenic variants in the *ATM* gene. Among these families, 13 had homozygous pathogenic variants, and 1 family had compound heterozygous variants. In total, 11 pathogenic variants were identified, with four of them being novel variants, including c.3320T > G, c.8050 C > T, c.3895del, and c.6453-2 A > G (as shown in Table 3). Among the pathogenic variants, 4 were missense variants and 1 was a splice variant. The remaining variants were categorized as truncating variants, including nonsense and frameshift variants, which accounted for 66.67% of the variants observed in our patients. Notably, a nonsense variant p.Arg2034Ter (c.6100 C > T) was found in two patients, one homozygous and one compound heterozygous patient. The distribution of *ATM* variants was similar among Fars and Azeri families, with 6 Fars patients and 4 Azeri families having *ATM* variants. The study also identified affected individuals from other ethnicities, as shown in Table 1.

#### Autosomal recessive spastic ataxia of charlevoix saguenay

In our study, twelve patients were found to have a variant in the *SACS* gene, which is responsible for ARSACS. Among these patients, ten of them had variants that were previously described in the literature [13]. The clinical and imaging profiles of all patients confirmed the diagnosis of ARSACS. We identified a total of ten variants in our patients, and two of them were novel, meaning they had not been reported before. One of the new patients had a missense variant, specifically c.11779G > C leading to p.Ala3927Pro. The second patient had two novel variants in a compound heterozygous genotype: c.10,625 A > G (p.Asp3542Gly) and c.12831_12841dupTCCTCTTTTCT (p.Ser4281PhefsTer31). The former variant was categorized as a Variant of Unknown Significance (VUS), while the latter was classified as a likely pathogenic variant.

Interestingly, the classic clinical triad of ARSACS, which typically includes progressive cerebellar ataxia, spasticity, and sensorimotor polyneuropathy, was not observed as a constant feature in all cases. However, all our patients did exhibit sensorimotor axonal-demyelinating neuropathy, and approximately half of them had spasticity and extensor plantar reflex. Brain magnetic resonance imaging revealed consistent findings in all patients, showing symmetric linear hypointensities in the pons, anterior superior cerebellar atrophy, and a hyperintense rim around the thalami on T2-weighted sequences. ARSACS was found to be more common among Azeri families, with six families having variants in the *SACS* gene, while only three Fars patients had possible causal variants in this gene (as indicated in Table 1; Fig. 1).

#### Conventional mutations in SCA genes: nine novel variants

Spinocerebellar ataxia (SCA) is a type of cerebellar ataxia characterized by progressive degeneration of the cerebellum, often accompanied by degenerative changes in other parts of the brain, including the brainstem, spinal cord, and even the peripheral nervous system. The condition is caused by ataxia genes with autosomal-dominant inheritance. While the most frequent causes of SCA are polyglutamine repeat expansion SCAs, a significant proportion of SCAs result from conventional mutations in SCA genes. In our study, we identified causal variants in known SCA genes among 8 patients, including *CWF19L1, SNX14, THG1L, VPS13D, RUBCN, GRID2, MME*, and *FAT2*. Among our patients, missense variants were particularly common, with 13 missense variants observed.

The ethnic distribution of the patients with SCA variants was as follows: 6 patients were of Fars ethnicity, 2 patients were Azeri population. Notably, gaze-evoked nystagmus was observed in half of all patients, providing an important clinical feature of the disease.

**Fig. 2:**
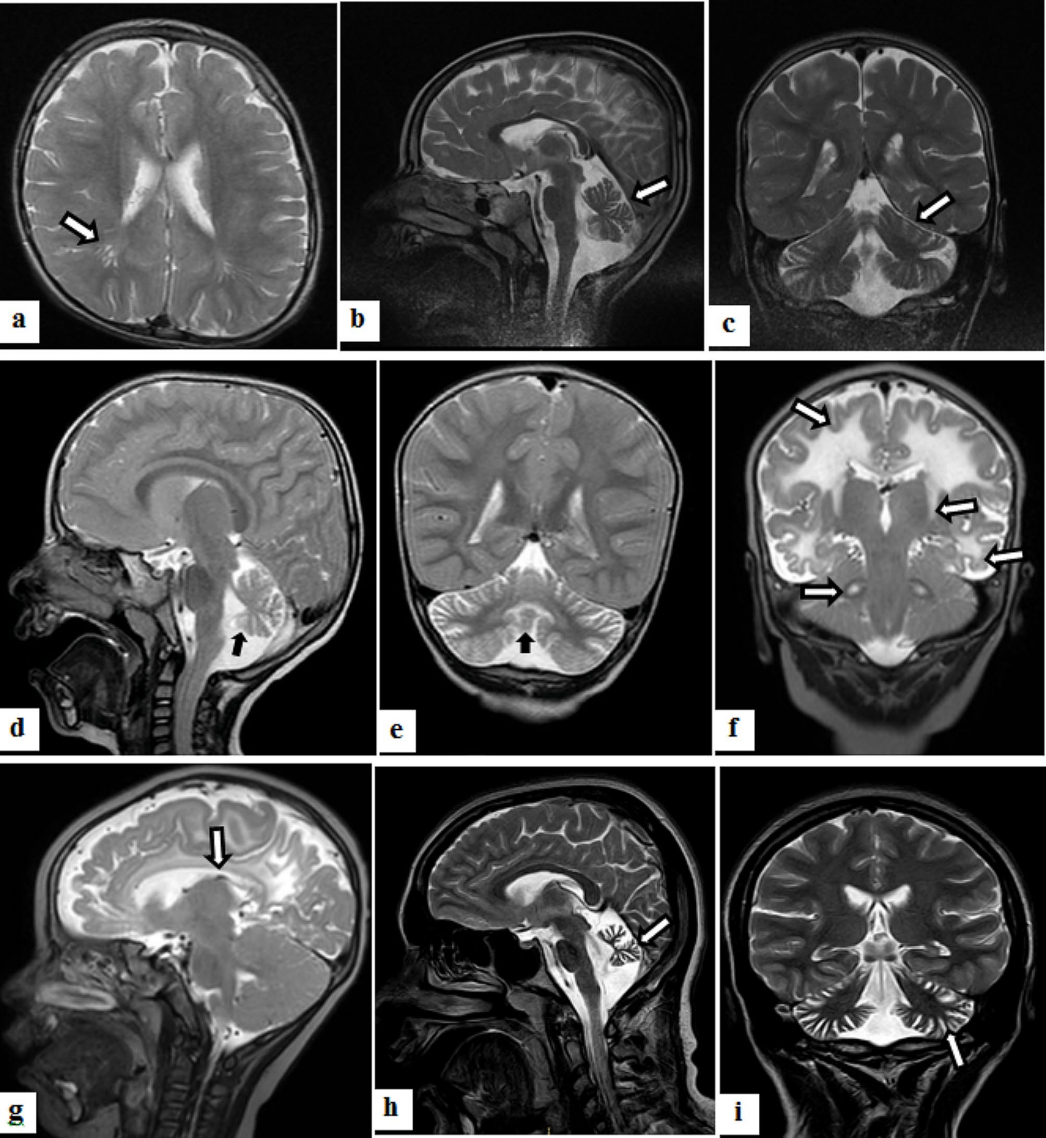
MRI of patients with c.574T > C (*CWF19L1*) variant (a-c), c.388G > A (*THG1L*) variant **(d,e)**, c.1781G > A (*DARS2*) variant **(f, g)** and c.398 A > G (*BRAT1*); **(a)** Axial T2-Weighted Brain MRI shows increased peri-vascular spaces. **(b)** Sagittal and **(c)** Coronal T2- Weighted Imaging shows cerebellar (prominently vermis) atrophy. **d** and **e**) T2-weighted sagittal and coronal images showing cerebellar atrophy. **f**) Coronal T2-Weighted Brain MRI is indicative of white matter, internal capsule and bilateral dentate involvement. **g**) Sagittal T2-Weighted view shows corpus callosum atrophy. **h**) Sagittal and **i**) Coronal T2-Weighted Brain MRI shows severe cerebellar atrophy

In this study, we identified seven novel variants in SCA patients, which were as follows: c.574T > C (*CWF19L1*), c.388G > A (*THG1L*), c.8305G > C (*VPS13D*), c.1721 C > G (*RUBCN*), c.1033 C > T (*GRID2*), c.2242 C > T (*MME*), and c.12913G > T (*FAT2*). Additionally, a reported variant c.1132 C > T (*SNX14*) was found. Clinical features of patient with c.574T > C (*CWF19L1*) were global developmental delay (GDD) (no speech, walking after 5 years), Autism spectrum disorder (ASD), epilepsy, hyperreflexia (upper and lower limbs), spasticity in upper and lower limbs, spastic gait and severe cognitive impairment. Brain MRI showed cerebellar (prominently vermis) atrophy and increased peri-vascular spaces in this patient (Fig. 2a-c). Patient with c.388G > A (*THG1L*) had neck holding at 6 months, sitting at 18 months, dependent walking (i.e., assisted walking) at 24 months, and a progressive gait problem and speech delay; other clinical findings were hyperreflexia (upper and lower limb), contracture, spasticity, dysarthria, cerebellar atrophy (2 years old) and chronic axonal type sensorimotor polyneuropathy. MRI showed cerebellar atrophy in this case (Fig. 2d,e). These findings contribute to a better understanding of the genetic basis of SCA and the diverse variants associated with this condition in different ethnic populations. Detailed clinical features of the SCA patients have been submitted elsewhere [26], providing further valuable insights into the presentation and characteristics of the disease in the study cohort.

#### Neuronal ceroid lipofuscinoses (NCLs): 2 novel variants

In our study, we focused on Neuronal ceroid lipofuscinosis 7 (CLN7), which is the most common type of NCL. Among the 7 NCL patients in our study, three of them had causal variants in the *MFSD8* gene. Additionally, the other four cases were attributed to variants in the *TPP1*, *CLN3*, *CLN5*, and *CLN6* genes. Two patients were from the Fars ethnicity, three were of Azeri descent, one patient was Kurdish, and the last one had Arab ethnicity. In the *MFSD8* gene, we identified two novel splice variants, which were classified as likely pathogenic according to ACMG2015 criteria. These variants are of particular interest as they have not been previously reported and may have a significant impact on the function of the *MFSD8* gene.

#### Mitochondrial ataxias

In our study, we identified variants in several genes associated with ataxias. Specifically, we found variants in the *POLG* gene (which is associated with a range of mitochondrial disorders, including ataxia), *BRAT1* gene (which is linked to a neurodevelopmental disorder with ataxia, intellectual disability, and other neurological features), *NARS2* gene (related to a condition known as *NARS2*-related disorders which can include ataxia among other symptoms), and *MT-ATP6* gene (which is associated with mitochondrial disorders that can manifest with ataxia and other neurological problems) in four patients. Additionally, we identified a homozygous Variant of Unknown Significance (VUS) in the *DARS2* gene in one family. This affected boy had normal head circumferences, weight and height at birth. His parents had a consanguineous marriage. The seizure occurred at the 2nd day. The diagnosis of hypothyroidism was made on the 40th day of his life. Other clinical features were hypotonia from early infancy, horizontal nystagmus and moderate cognitive decline. His younger sister showed autism, epilepsy, Developmental delay and optic nerve atrophy. His Coronal T2-Weighted Brain MRI was indicative of white matter internal capsule and bilateral dentate involvements and corpus callosum atrophy (Fig. 2f,g).

One novel variant was found in an Azeri patient in our study. c.398 A > G in *BRAT1* gene was found in a girl with GDD; she recently walked supported in a ataxic wide-based gait manner (unsupported walking was not achieved), independent sitting achieved at age 1–2 years, language contained some limited simple word, declined cognitive function. In this patient, severe cerebellar atrophy was observed (Fig. 2h,i). Other clinical features of this patient were myopia, photo sensitivity and moderate cognitive decline. Furthermore, under the treatable ataxias subheading, three patients were found to have variants in the *COQ8A* gene, which is associated with Coenzyme Q10 deficiency, a condition that can be responsive to treatment with CoQ10 supplementation.

#### Congenital ataxias

In our study, we focused on a group of ataxias characterized by cerebellar structural anomalies in motor development and stable non-progressive ataxias. We identified two frameshift variants in the *AHI1* and *TMEM237* genes, leading to Joubert syndrome types 3 and 14, respectively. Additionally, one patient had a missense variant in the *CEP120* gene. Furthermore, we discovered a novel frameshift variant in the *ATCAY* gene in a patient from a consanguineous marriage, associated with Cayman ataxia [27]. Patients with Cayman ataxia typically present with hypotonia, psychomotor delay, and non-progressive cerebellar dysfunction.

#### Miscellaneous disorders

In our study, we encountered several families with ataxias caused by variants in genes that were not previously classified or categorized under specific known ataxia-related genes. We referred to these families as “uncategorized genes” due to the lack of clear associations with established ataxia-related genes. The specific genes identified in these families and their associated conditions are as follows:

1. *HEXA* gene: Associated with Tay-Sachs disease, a rare neurodegenerative disorder.
2. *KIF1C* gene: Linked to Autosomal Recessive Spastic Ataxia-2 (SPAX2).
3. *FIG4* gene: Associated with Charcot-Marie-Tooth Type 4 J (CMT4J), a type of hereditary motor and sensory neuropathy.
4. *SIL1* gene: Linked to Marinesco-Sjogren syndrome, which is characterized by cerebellar ataxia, cataracts, and muscle weakness.
5. *ERLIN2* genes: Associated with Spastic Paraplegia 18, a type of hereditary spastic paraplegia.
6. *ELP2* gene: Linked to Intellectual Developmental Disorder-58.
7. *ADD3* gene: Associated with Spastic Quadriplegic Cerebral Palsy.
8. *RNASET2* gene: Linked to Cystic Leukoencephalopathy without Megalencephaly, a rare neurological disorder characterized by abnormal white matter in the brain.
9. *SAMD9* gene: associatd with MIRAGE syndrome.
10. *WDR81* gene: associated with cerebellar hypoplasia and quadrupedal locomotion 2.

The identification of these variants in the respective genes expands our understanding of the genetic causes of ataxias and underscores the genetic heterogeneity of this group of disorders. It also highlights the importance of ongoing research and genetic analysis to further classify and characterize the underlying genetic basis of ataxias with unidentified gene associations.

**Table 2:** the studied patients and their responsible genes and variants in our study

patient no	Family No.	Gene	Variant Coordinates hg38	NM#	Nucleotide change	AA change	Novel/reported	Reference
1	1	*PLA2G6*	Chr22:g38115658	NM_003560.4	c.1903 C > T	p.Arg635Ter	Reported	PMIDs:32,357,911, 29,482,223, 16,783,378, 30,340,910, 25,164,370, 22,934,738
2	2	*PLA2G6*	Chr22:g38140111	NM_003560.4	c.668 C > T	p.Pro223Leu	Reported	PMID: 29,454,663
3	3	*PLA2G6*	Chr22:g38113581	NM_003560.4	c.2108T > A	p.Val703Glu	Novel	-
4	4	*PLA2G6*	Chr22:g38140111	NM_003560.4	c.668 C > T	p.Pro223Leu	Reported	PMID: 29,454,663
5	5	*PLA2G6*	Chr22:g38132946	NM_003560.4	c.962T > C	p.Leu321Pro	Novel	-
		*PLA2G6*	Chr22:g38132869		c.1039G > A	p.Gly347Arg	Reported	PMIDs:31,496,990, 27,196,560, 26,196,026, 16,783,378,
6	6	*PLA2G6*	Chr22:g38115589	NM_003560.4	c.1972 A > G	p.Asn658Asp	Novel	-
7	7	*PLA2G6*	Chr22:g38135012	NM_003560.4	c.865_869dup	p.Leu291AlafsTer16	Novel	-
8	8	*PLA2G6*	Chr22:g38140106	NM_003560.4	c.673 C > T	p.His225Tyr	Reported	PMID: 24,130,795
9	9	*PLA2G6*	Chr22:g38120879	NM_003560.4	c.1622 A > C	p.Tyr541Ser	Novel	-
10	10	*PLA2G6*	Chr22:g38112212	NM_003560.4	c.2370T > G	p.Tyr790Ter	Reported	PMIDs:30,868,093, 20,886,109, 32,357,911, 29,913,018, 30,293,248, 20,495,927
11	11	*PLA2G6*	Chr22:g38115589	NM_003560.4	c.1972 A > G	p.Asn658Asp	Novel	-
12	12	*PLA2G6*	Chr22:g38112559	NM_003560.4	c.2221 C > T	p.Arg741Trp	Reported	PMIDs: 27,196,560, 20,886,109, 31,516,627, 30,713,958
13	13	*PLA2G6*	Chr22:g38123259	NM_003560.4	c.1428-1G > A		Reported	PMID:35,083,005
14	14	*PLA2G6*	Chr22:g38115612	NM_003560.4	c.1949T > C	p.Phe650Ser	Reported	PMID:35,083,005
15	15	*PLA2G6*	Chr22:g38120889	NM_003560.4	c.1612 C > A	p.Arg538Ser	Reported	PMID:30,363,890
16	16	*PLA2G6*	Chr22:g38132923	NM_003560.4	c.985 C > T	p.Arg329Cys	Reported	PMID:27,196,560
17	17	*PLA2G6*	Chr22:g38126370	NM_003560.4	c.1427 + 1G > C		Novel	-
18	18	*PLA2G6*	Chr22:g38115546	NM_003560.4	c.2015 A > T	p.Asn672Ile	Novel	-
19	19	*PLA2G6*	Chr22:g38132923	NM_003560.4	c.985 C > T	p.Arg329Cys	Reported	PMID:27,196,560
20	20	*CWF19L1*	Chr10:g100253470	NM_018294.6	c.574T > C	p.Tyr192His	Novel	-
21	21	*SNX14*	Chr6:g85543737	NM_153816.6	c.1132 C > T	p.Arg378Ter	Reported	PMID:33,193,593, 25,848,753
22	22	*THG1L*	Chr5:g157734595	NM_017872.5	c.388G > A	p.Ala130Thr	Novel	-
23	23	*VPS13D*	Chr1:g12333243	NM_015378.4	c.8305G > C	p.Glu2769Gln	Novel	-
24	24	*RUBCN*	Chr3:g197693780	NM_014687.4	c.1721 C > G	p.Ser574Ter	Novel	-
25	25	*GRID2*	Chr4:g 93,224,683	NM_001510.4	c.1033 C > T	p.Arg345Ter	Novel	-
26	26	*MME*	Chr3:g155180448	NM_001354642.1	c.2242 C > T	p.Arg748Trp	Novel	-
27	27	*FAT2*	Chr5:g151505702	NM_001447.3	c.12913G > T	p.Ala4305Ser	Novel	-
28	28	*ATM*	Chr11:g108321299	NM_000051.4	c.6453-2 A > G		Novel	-
29	29	*ATM*	Chr11:g108315863	NM_000051.4	c.6047 A > G	p.Asp2016Gly	Reported	PMID:9,887,333, 11,826,030, 24,825,865, 11,826,026, 11,826,029
30	30	*ATM*	Chr11:g108279526	NM_000051.4	c.3320T > G	p.Leu1107Ter	Novel	-
31	31	*ATM*	Chr11:g108244954	NM_000051.4	c.829G > T	p.Glu277Ter	Reported	PMID:32,962,506, 32,091,409
32	32	*ATM*	Chr11:g108316015	NM_000051.4	c.6100 C > T	p.Arg2034Ter	Reported	PMID:11,505,391, 29,731,985
33	33	*ATM*	Chr11:g108316015	NM_000051.4	c.6100 C > T	p.Arg2034Ter	Reported	PMID:11,505,391, 29,731,985
34	34	*ATM*	Chr11:g108335008	NM_000051.4	c.8050 C > T	p.Gln2684Ter	Novel	-
35	35	*ATM*	Chr11:g108365138	NM_000051.4	c.6100 C > T	p.Tyr2969Ter	Reported	PMID:11,505,391, 29,731,985
		*ATM*	Chr11:g108227691	NM_000051.4	c.67 C > T	p.Arg23Ter	Reported	PMID:26,506,520
36	36	*ATM*	Chr11:g108307928	NM_000051.4	c.5712dup	p.Ser1905IlefsTer25	Reported	PMID:18,321,536
37,38	37	*ATM*	Chr11:g108317432	NM_000051.4	c.6259delG	p.Glu2087LysfsTer9	Reported	PMID:32,095,276
39	38	*ATM*	Chr11:g108244954	NM_000051.4	c.829G > T	p.Glu277Ter	Reported	PMID:32,962,506, 32,091,409
40	39	*ATM*	Chr11:g108315863	NM_000051.4	c.6047 A > G	p.Asp2016Gly	Reported	PMID:9,887,333, 11,826,030, 24,825,865, 11,826,026, 11,826,029
41	40	*ATM*	Chr11:g108284374	NM_000051.4	c.3895del	p.Ala1299ProfsTer50	Novel	-
42	41	*ATM*	Chr11:g108330234	NM_000051.4	c.7328G > A	p.Arg2443Gln	Reported	PMID:31,740,029, 31,754,145, 26,630,574, 27,175,599
43	42	*SACS*	Chr13:g23333063	NM_014363.6	c.10,813 A > T	p.Lys3605Ter	Reported	PMID: 35,731,353
44	43	*SACS*	Chr13:g23334010	NM_014363.6	c.9866 C > G	p.Ser3289Ter	Reported	PMID: 35,731,353
45	44	*SACS*	Chr13:g23335744	NM_014363.6	c.8132 C > A	p.Ser2711Ter	Reported	PMID: 35,731,353
46	45	*SACS*	Chr13:g23341436	NM_014363.6	c.2439_2440delAT	p.Val815GlyfsTer4	Reported	PMID: 35,731,353
47	46	*SACS*	Chr13:g23340595	NM_014363.6	c.3281dupA	p.Asn1094LysfsTer18	Reported	PMID: 35,731,353
48	47	*SACS*	Chr13:g23340449	NM_014363.6	c.3427 C > T	p.Gln1143Ter	Reported	PMID: 35,731,353
49	48	*SACS*	Chr13:g23335160	NM_014363.6	c.8716 C > T	p.Arg2906Ter	Reported	PMID: 35,731,353
50	49	*SACS*	Chr13:g23336372	NM_014363.6	c.7504 C > T	p.Arg2502Ter	Reported	PMID: 35,731,353
51	50	*SACS*	Chr13:g23340183	NM_014363.6	c.3695_3697del	p.Val1232del	Reported	PMID: 35,731,353
52	51	*SACS*	Chr13:g23332097	NM_014363.6	c.11779G > C	p.Ala3927Pro	Reported	PMID: 35,731,353
53	52	*SACS*	Chr13:g23332097	NM_014363.6	c.11779G > C	p.Ala3927Pro	Reported	PMID: 35,731,353
54	53	*SACS*	Chr13:g.23,907,390	NM_014363.6	c.10,625 A > G	p.Asp3542Gly	Novel	
			Chr13:g.23,905,173	NM_014363.6	c.12831_12841dupTCCTCTTTTCT	p.Ser4281PhefsTer31	Novel	
55	54	*APTX*	Chr9:g32974539	ENST00000379817.7	c.793 A > T	p.Ser265Cys	Novel	-
56,57	55	*APTX*	Chr9:g32984821	ENST00000379817.7	c.582del	p.Lys194AsnfsTer20	Novel	-
58	56	*APTX*	Chr9:g32984667	ENST00000379817.7	c.734G > A	p.Arg245His	Novel	-
59	57	*APTX*	Chr9:g32984842	ENST00000379817.7	c.559 C > T	p.Gln187Ter	Reported	PMID:29,356,829
60	58	*APTX*	Chr9:g32987650	NM_001195248.2	c.376delA	p.Arg126GlyfsTer26	Novel	-
61	59	*COQ8A*	Chr1:g226983633	NM_020247.5	c.1162G > A	p.Gly388Ser	Novel	-
62	60	*COQ8A*	Chr1:g226982107	NM_020247.5	c.811 C > T	p.Arg271Cys	Reported	PMID:24,218,524, 30,637,285, 32,337,771, 24,164,873, 29,255,295, 29,915,382
63	61	*COQ8A*	Chr1:g227169811	NM_020247.5	c.814G > T	p.Gly272Cys	Reported	PMID:35,275,351
64,65	62	*TTPA*	Chr8:g63061291	NM_000370.3	c.798del	p.Glu267LysfsTer27	Novel	-
66	63	*NPC2*	Chr14:g74484506	NM_001375440.1	c.272 A > T	p.Asp91Val	Novel	-
67	64	*NPC2*	Chr14:74484506	NM_006432.5	c.272 A > T	p.Asp91Val	Novel	-
68	65	*MFSD8*	Chr4:g127965071	NM_152778.3	c.62 + 1G > A		Novel	-
69	66	*MFSD8*	Chr4:g127930787	NM_001371596.1	c.894T > G	p.Tyr298Ter	Reported	PMID:17,564,970
70,71	67	*MFSD8*	Chr4:g127943750	NM_001371596.1	c.439 + 2T > C	-	Novel	-
72	68	*TPP1*	Chr11:g6615170	NM_000391.4	c.1425 + 1G > T		Novel	-submitted
73	69	*CLN3*	Chr16:g28477598	NM_001286110.2	c.1073_1074insAGAGAAATGAATGAGCCTACAGATGATAGGATGTGGTGTT	p.Cys359GlufsTer3	Novel	-
74	70	*CLN6*	Chr15:g68211685	NM_017882.3	c.476 C > T	p.Pro159Leu	Reported	PMID:30,285,654, 19,201,763
75	71	*CLN5*	Chr13:g76992159	ENST00000377453.9	c.61 C > T	p.Arg21Trp	Reported	PMID:22,727,047, PMID:21,990,111
77	72	*POLG*	Chr15:g89318535	ENST00000268124.11	c.3482 + 6 C > T		Novel	-
78	73	*BRAT1*	Chr7:g2544941	ENST00000340611.9	c.398 A > G	p.His133Arg	Novel	-
79	74	*MT-ATP6*	ChrM:8993	ENST00000361899.2	c.467T > G	p.Leu156Arg	Reported	PMID: 1,539,598
80	75	*DARS2*	Chr1:g173857548	NM_018122.5	c.1781G > A	p.Gly594Glu	Novel	-
81,82	76	*NARS2*	Chr11:g78559588	NM_024678.6	c.545T > A	p.Ile182Lys	Reported	PMID:34,374,940
83	77	*TMEM237*	Chr2:g201632053	NM_001044385.3	c.550dup	p.Ser184LysfsTer8	Novel	-
84	78	*AHI1*	Chr6:g135463145	NM_001134831.2	c.910dup	p.Thr304AsnfsTer6	Reported	PMID:26,541,515
85	79	*CEP120*	Chr5:123377502	NM_001375405.1	c.2230 C > T	p.Arg744Cys	Novel	-
86	80	*ATCAY*	Chr19(hg38):g3913774	NM_033064.5	c.883_884del	p.Lys295AspfsTer52	Reported	[27]
87	81	*GAN*	Chr16:g81363869	NM_022041.4	c.1162delC	p.Leu388Ter	Reported	[28]
88,89	82	*GAN*	Chr16:g81354492	NM_022041.4	c.370T > A	p.Phe124Ile	Reported	[28]
90	83	*SAMD9*	Chr7:g93101540	NM_001193307.1	c.4558G > T	p.Glu1520Ter	Novel	-
91	84	*HEXA*	Chr15:g72345461	NM_000520.6	c.1511G > A	p.Arg504His	Reported	PMID:16,088,929, 31,367,523, 29,482,223
92	85	*KIF1C*	Chr17:g5020864	NM_006612.6	c.1996G > T	p.Glu666Ter	Novel	-
93	86	*RNASET2*	Chr6:g166938996	NM_003730.6	c.345G > A	p.Trp115Ter	Novel	-
94	87	*FIG4*	Chr6:g109776937	NM_014845.6	c.1766 A > G	p.Asp589Gly	Novel	-
95,96	88	*SIL1*	Chr5:g138951290	NM_022464.5	c.910 C > T	p.Gln304Ter	Novel	-
97	89	*ADD3*	Chr10:g110122249	NM_016824.5	c.1100G > A	p.Gly367Asp	Reported	PMID:23,836,506, 28,492,530, 30,369,941, 28,042,670, 27,391,121
98	90	*WDR81*	Chr17:g1726544	NM_001163809.2	c.1585 C > G	p.Arg529Gly	Novel	-
99	91	*ERLIN2*	Chr8:37744568	NM_007175.8	c.299-3 C > T	-	Novel	-
100	92	*ELP2*	Chr18:36159780	NM_018255.4	c.1580G > A	p.Ser527Asn	Novel	-

Five patients were homozygous for Anemia Fancony expansion

**Table 3:** genes, novel variants hg38 and their phenotypes and in silico predictions

No.	Gene	Nucleotide variant	AA change	Predictions				ACMG classification
				MutationTaster	SIFT	Polyphen-2	CADD score GRCh38-v1.6	
1	*APTX*	c.793 A > T	p.Ser265Cys	DC	D	NA	27.7	VUS
2	*APTX*	c.582del	p.Lys194AsnfsTer20	NA	NA	NA	NA	Pathogenic
3	*APTX*	c.734G > A	p.Arg245His	DC	T	NA	22.5	VUS
4	*APTX*	c.376delA	p.Arg126GlyfsTer26	NA	NA	NA	NA	Pathogenic
5	*ATM*	c.3320T > G	p.Leu1107Ter	DC	NA	NA	34	Pathogenic
6	*ATM*	c.8050 C > T	p.Gln2684Ter	DC	NA	NA	38	Pathogenic
7	*ATM*	c.3895del	p.Ala1299ProfsTer50	NA	NA	NA	NA	Pathogenic
8	*ATM*	c.6453-2 A > G	-	DC	NA	NA	34	Pathogenic
9	*BRAT1*	c.398 A > G	p.His133Arg	DC	D	PD	24.8	VUS
10	*CEP120*	c.2230 C > T	p.Arg744Cys	DC	T	B	25.3	VUS
11	*CLN3*	c.1073_1074insAGAGAAATGAATGAGCCTACAGATGATAGGATGTGGTGTT	p.Cys359GlufsTer3	NA	NA	NA	NA	Pathogenic
12	*COQ8A*	c.1162G > A	p.Gly388Ser	DC	D	PD	35	VUS
13	*CWF19L1*	c.574T > C	p.Tyr192His	DC	D	PD	27.9	VUS
14	*DARS2*	c.1781G > A	p.Gly594Glu	DC	T	PD	24.8	VUS
15	*ELP2*	c.1580G > A	p.Arg527Gln	DC	T	B	10.43	VUS
16	*ERLIN2*	c.299-3 C > T	-	NA	NA	NA	16.97	VUS
17	*FAT2*	c.12913G > T	p.Ala4305Ser	DC	D	PD	24.7	VUS
18	*FI.G4*	c.1766 A > G	p.Asp589Gly	DC	T	B	23.5	VUS
19	*FTL*	c.325 C > T	p.Gln109Ter	DC	NA	NA	37	Likely pathogenic
20	*GRID2*	c.1033 C > T	p.Arg345Ter	DC	NA	NA	35	Pathogenic
21	*KIF1C*	c.1996G > T	p.Glu666Ter	DC	NA	NA	50	Pathogenic
22	*MFSD8*	c.62 + 1G > A	-	DC	NA	NA	33	Likely pathogenic
23	*MFSD8*	c.439 + 2T > C	-	DC	NA	NA	33	Likely pathogenic
24	*MME*	c.2242 C > T	p.Arg748Trp	DC	D	PD	28.1	VUS
25	*NPC2*	c.272 A > T	p.Asp91Val	DC	D	PD	27.8	VUS
26	*PLA2G6*	c.2108T > A	p.Val703Glu	DC	D	PD	29.4	VUS
27	*PLA2G6*	c.962T > C	p.Leu321Pro	DC	D	PD	26.9	Likely pathogenic
28	*PLA2G6*	c.1972 A > G	p.Asn658Asp	DC	D	PD	28.3	Likely pathogenic
29	*PLA2G6*	c.865_869dup	p.Leu291AlafsTer16	NA	NA	NA	NA	Pathogenic
30	*PLA2G6*	c.1622 A > C	p.Tyr541Ser	DC	D	PD	28.3	Likely pathogenic
31	*PLA2G6*	c.1427 + 1G > C	-	DC	NA	NA	33	Pathogenic
32	*PLA2G6*	c.2015 A > T	p.Asn672Ile	DC	D	PD	29.8	VUS
33	*POLG*	c.3482 + 6 C > T	-	NA	NA	NA	1.24	VUS
34	*RNASET2*	c.345G > A	p.Trp115Ter	DC	NA	NA	36	Pathogenic
35	*RUBCN*	c.1721 C > G	p.Ser574Ter	DC	NA	NA	38	Pathogenic
36	*SACS*	c.10,625 A > G	p.Asp3542Gly	DC	D	PD	27.3	VUS
37	*SACS*	c.12831_12841dupTCCTCTTTTCT	p.Ser4281PhefsTer31	NA	NA	NA	NA	Likely pathogenic
38	*SAMD9*	c.4558G > T	p.Glu1520Ter	DC	NA	NA	34	Likely pathogenic
39	*SIL1*	c.910 C > T	p.Gln304Ter	DC	NA	NA	41	Pathogenic
40	*THG1L*	c.388G > A	p.Ala130Thr	DC	T	B	22.3	VUS
41	*TMEM237*	c.550dup	p.Ser184LysfsTer8	NA	NA	NA	NA	Pathogenic
42	*TPP1*	c.1425 + 1G > T	-	DC	NA	NA	34	Likely pathogenic
43	*TTPA*	c.798del	p.Glu267LysfsTer27	NA	NA	NA	NA	Likely pathogenic
44	*VPS13D*	c.8305G > C	p.Glu2769Gln	DC	NA	PD	27.9	VUS
45	*WDR81*	c.1585 C > G	p.Arg529Gly	DC	D	PD	23.2	VUS

DC: disease causing; PD: probably damaging; D: Damaging; T: tolerated; N: Neutral; B: benign

## Discussion

Hereditary cerebellar ataxias represent a diverse group of rare genetic disorders with various modes of inheritance. Despite the rarity of these disorders, there has been a lack of comprehensive genetic studies focusing on the Iranian population. To address this gap, we conducted a genetic study on patients with hereditary ataxias in Iran, aiming to identify common genes, variants, and proteins involved in the pathways within cerebellar cells specific to this population. Our study included 105 patients from 97 families with hereditary ataxias, and we observed the following frequencies for different subtypes:

(a) 19 families with Infantile Neuroaxonal Dystrophy (INAD); (b) 16 families with Treatable ataxias; (c) 14 families with AT; (d) 12 families with ARSACS; (e) 8 families with Spinocerebellar Ataxias (SCAs); (f) 7 families with Neuronal Ceroid Lipofuscinoses (NCLs); (g) 5 families with Mitochondrial ataxias; (h) 4 families with congenital ataxias; (i) 2 families with Giant Axonal Neuropathy (GAN); and (j) 10 families with other rare and less characterized disorders.

It is worth noting that the Fars and Azeri subpopulations in our country are larger than other subpopulations, which is why most of the patients in our study come from these populations. The distribution of disorders is shown in Table 1. Among the Fars cohort, INADs, SCAs, Treatable ataxias, and AT account for more than 66% of ataxic patients, while ARSAC, SCAs, Treatable ataxias, and AT are observed in approximately 60% of Azeri patients. These findings highlight the genetic heterogeneity of hereditary cerebellar ataxias in the Iranian population. Our study provides valuable insights into the prevalence of different subtypes of ataxias in this specific ethnic group and contributes to the understanding of the underlying genetic basis of these disorders. The identification of common genes and variants in this population can lead to improved diagnosis, management, and potential targeted therapies for patients with hereditary ataxias. The distribution of disorders among various subpopulations can be attributed to specific reasons. For example, the founder effect may be observed in some subpopulations, leading to a high frequency of a particular disorder. Traditional customs still exist in many Iranian ethnicities, and the rate of consanguineous and intragroup marriages is higher than in European countries. These factors may contribute to an increased frequency of certain diseases.

Our research adds to the growing body of knowledge on ataxias and paves the way for further investigations to unravel the molecular mechanisms and genetic pathways involved in these complex disorders.

The results of a 4-year follow-up for 25 Iranian patients with treatable ataxia showed that early detection of treatable ataxia, close observation, and follow-up [26] could benefit patients. It is known that the treatment of cerebellar ataxias is still supportive and symptom-dependent, but a limited number of progressive ataxic forms may respond to disease-specific treatments if diagnosed early. Some of these inherited forms include Ataxia with vitamin E deficiency (AVED), Abetalipoproteinemia, Ataxia with oculomotor apraxia (*APTX*), Cerebrotendinous xanthomatosis, Niemann–Pick disease (type C), Autosomal recessive cerebellar ataxia due to coenzyme Q10 deficiency, Refsum’s disease, Glucose transporter type 1 deficiency, Friedreich’s ataxia, and Episodic ataxia type 2. Among Iranian populations, INADs, 16 SCAs, and treatable ataxias account for 50% of all forms of ataxias and are the most common.

INAD is apparently a common disorder among the Fars ethnicity but rare among other ethnicities, while ARSAC is more prevalent in the Azeri subpopulation. The most common type of ARSACS variants is responsible for 13%, followed by *SPG7* (10%), AT (7%), *AOA2* (7%), *RFC1* (7%), *COQ8A* (5%), *POLG* (4%), *AOA1* (3%), and *ANO10* (3%) [9]. In another study on an eastern Asian cohort, variants of ten genes were determined in 54 Chinese patients. Four of these genes accounted for 37.0% of the positive patients [29], with *SACS*, *ADCK3*, and *SETX* variants responsible for 9, 6, and 5 Chinese patients, respectively. Frequencies of different types of ataxias vary among Iranian subpopulations (Table 1). Additionally, some discrepancies are observed in the prevalence of involved genes in our population compared to other cohorts. For instance, *PLA2G6* variants were detected in 19 families. However, previous studies did not find its variants in 110 Algerian families and European descendants [9, 30, 31]; this may be due to that INAD (*PLA2G6* ) was not classified as an ARCA in these previous studies as ataxia is not the main feature of this condition, therefore these patients were probably not recruited, which explain the absence of INAD patients in these cohorts. Similarly, while *SPG7* is responsible for about 10% of European patients [9], none of the Chinese patients [29] and our patients showed *SPG7* variants. Some variants and genes are common among specific populations and their high frequencies may be due to founder effects [32, 33]; for example, a large deletion in the *GJB6* gene delta (*GJB6*-D13S1830) causes hearing loss in many populations but is not found in some populations [34].

A multicenter study of autosomal recessive cerebellar ataxias (ARCA) across South America from 11 large ataxia centers revealed that in these regions, over 40% of ataxia cases had a positive molecular diagnosis for ARCA. Interestingly, FA was observed in 57% of these cases [35]. In the largest European ARCA frequency study, 59% of 677 patients showed a genetic variant [9]. FA is characterized by sensory axonal neuropathy, absent lower limb tendon reflexes, scoliosis, hypertrophic cardiomyopathy, pes cavus, and diabetes mellitus. In our study, the most common neurological findings of the FRDA patients were sensory neuropathy and dysarthria. Additionally, scoliosis was another common clinical presentation observed in three of the cases. However, brain MRI scans of all FRDA patients showed no abnormal features; the scans were unremarkable. These findings contribute to our understanding of the genetic basis of Friedreich’s ataxia in the study population and highlight the importance of GAA triplet repeat expansions within the frataxin gene as a key diagnostic marker. The successful treatment of these patients with the FDA-approved drug Omexavalone underscores the potential therapeutic benefits of targeted treatments for FRDA.

In over 95% of cases, Friedreich’s ataxia (FA) occurs due to biallelic expansion of the GAA repeat in intron 1 of *FXN*, which encodes frataxin—a mitochondrial chaperone involved in iron-sulfur biogenesis and heme biosynthesis. The remaining patients are compound heterozygotes with one single repeat expansion and a point mutation. *FXN* expansion as the most common mutation in was observed in about 5% of our patients, while it accounted for 28% and 44.54% of patients from Algeria and eastern France, respectively. However, only 2 out of 96 patients from Finland showed *FXN* expansions [36]. FA has been reported as a rare disorder in eastern Asia [37]. It is possible that most of the patients referred to our ataxia clinic had unsolved progressive ataxia, and confirmed cases of FA in our country might not have been referred for registry.

There are discrepancies in the frequencies of certain mutations as well. For instance, *TTPA* gene variants have been reported in 14.5% of patients from eastern France, but only one of our patients showed *TTPA* variants. Interestingly, *SETX* variants (associated with AOA2) were not found in our patients, while this gene was responsible for 2, 12, and 12 families in the Irish, Algerian, and eastern French studies, respectively [30, 31, 38]. On the other hand, AOA1 (*APTX*) and *SIL1* showed the same frequency between French patients and Iranian families, whereas only about 0.5% of Irish patients were affected by AOA1. In Traschutz’s study, about 3% and 7% of European patients showed a variant in the *APTX* and *SYNE1* genes, respectively [9]. The majority of these patients with ataxia are observed among the Fars and Azeri ethnicities. In this study, four novel pathogenic variants are described, with three of them leading to a truncated protein and the fourth being a splice variant. Along with ataxia, individuals with this condition may exhibit oculomotor apraxia, involuntary jerking movements, muscle wasting in their hands and feet, and neuropathy. As before mentioned, among five *APTX*-related families in our study, three novel variants were found; these findings provide further insights into the genetic basis of Ataxia with Oculomotor Apraxia Type 1 in the study population. The identification of novel variants adds to our understanding of the genetic heterogeneity of this condition and may have implications for diagnosis, prognosis, and potential targeted treatments in the future.

AT is a rare inherited disorder caused by pathogenic variants in the *ATM* gene, which affects the nervous system, immune system, and other body systems. The product of the *ATM* gene plays a crucial role in DNA repair. As shown in Tables 1 and 2, findings of this study provide important insights into the genetic basis of AT in the study population and highlight the prevalence of pathogenic variants in the *ATM* gene, with a considerable proportion being novel variants. The identification of various variant types emphasizes the genetic heterogeneity of AT and its impact on different ethnic groups. AT is also prevalent among our population, affecting more than 10% of Iranian patients with ataxia. However, Algerian and eastern French studies did not report anything about the *ATM* gene in their patient samples; although this form of ataxia is present in Algeria as it was reported by Tazir et al., 2009 [39]. In an Irish study, AT was observed in 4 out of 196 patients (2%) [38].

ARSACS affects approximately 10% of ataxic patients in our population, whereas it affects 13% of European patients [9]. In our ARSACS patients, we identified eight pathogenic variants and one Variant of Unknown Significance (VUS) sequence variant. Among the identified VUS variants, one of them has not been reported previously. To further confirm the impact of the identified VUS variant, we utilized the MetaDome server (data not shown), which showed that the variant was located in intolerant regions of the *SACS* in protein encoded by the *SACS* gene. This additional analysis adds weight to the potential pathogenicity of the VUS variant in ARSACS. These findings expand our knowledge of the genetic landscape of ARSACS and provide insights into the clinical and imaging features of the disease in the studied population. The identification of novel variants emphasizes the genetic heterogeneity of ARSACS and highlights the importance of considering the ethnic distribution of variants in different populations. Interestingly, only 4–5% of affected families in Algeria and eastern France have mutations in the *SACS* gene [31], while this frequency seems to be only 1% among Irish patients [38]. Such discrepancies in the distribution of genes and variants in patients from various ethnicities have been frequently reported.

NCLs (Neuronal ceroid lipofuscinoses) are a group of rare autosomal recessive disorders characterized by myoclonic epilepsy, psychomotor delay, ataxia, progressive loss of vision, and early death. Regarding the ethnic distribution of NCL patients, NCLs were observed among different ethnicities including Fars (2 patients), Azeri (3 cases), Kurdish (1 patient) and Arab (1 case) in our study. This finding underscores the genetic heterogeneity of NCL and the importance of considering different ethnic backgrounds in genetic studies. The identification of specific causal variants in NCL patients is critical for accurate diagnosis and potential targeted treatments in the future. These findings contribute to our understanding of the genetic basis of NCL and highlight the significance of genetic testing in the clinical management of affected individuals.

Congenital ataxia patients may also exhibit additional symptoms such as hypotonia, apnea, apraxia, and learning disabilities. One prominent example within this group is Joubert syndrome and related disorders, which are mainly caused by pathogenic variants in genes encoding ciliary proteins. These findings contribute to our understanding of the genetic basis of ataxias with cerebellar structural anomalies and non-progressive features. The identification of specific causal variants in genes associated with these conditions is crucial for accurate diagnosis and potentially targeted therapies in the future. The study of rare genetic disorders like Joubert syndrome and related conditions is essential for advancing our knowledge of ciliary biology and its role in normal neurological development and function.

The types and frequencies of variants in our study differ from those observed in Algerian and European patients [9, 30]. For instance, the c.744delA (p.Glu249Asnfs*15) variant, reported in 19 patients (16 families) in the Algerian cohort study [31], may indicate a possible founder effect in that population. In our study, we identified the c.798del variant in only one family, making it a novel and rare variant. Some variants with high frequencies in our population have been attributed to a founder effect [31, 33].

## Conclusion

This study represents the first investigation into the genetics of ARCA in a heterogeneous population in the Middle East. The diverse ethnicities across various geographic regions of Iran might also be representative of neighboring countries and could have common ancestry. Consequently, the distribution and frequencies of genetic variants in these ethnicities may offer insights into the populations of these neighboring regions. Notably, the frequencies and types of involved genes in our populations differ somewhat from those observed in other populations.

In Iran, common types of ataxia include INADs, SCAs, Treatable ataxias, AT, and ARSAC. These findings suggest that ataxia exhibits diverse genetic characteristics among different populations in the Middle East, highlighting the importance of studying the genetic variations within specific ethnic groups to gain a comprehensive understanding of the genetic basis of ataxia in the region. These findings highlight the genetic heterogeneity of ataxias and demonstrate the importance of targeted genetic testing in diagnosing and managing patients with various forms of the disorder. Additionally, the identification of specific genetic variants can inform treatment decisions, especially in cases where treatable forms of ataxia are present.

## Data Availability

No datasets were generated or analysed during the current study.

## Abbreviations

AGI: Ataxia Global Initiative
AOA: Ataxia with oculomotor apraxia
ARCA: Autosomal recessive cerebellar ataxia
ARSACS: Autosomal recessive spastic ataxia of Charlevoix-Saguenay
AT: Ataxia telangiectasia
AVED: Ataxia with Vitamin E Deficiency
BAM: Binary Alignment Map
CMC: Children’s Medical Center
CoQ10: Coenzyme Q10
CRF: Case Report Form
EDX: Electrodiagnostic exams
EOAs: Early-onset ataxias
FA: Friedreich ataxia
GAN: Giant Axonal Neuropathy
GATK: Genome Analysis Toolkit Haplotypecaller
GDD: Global developmental delay
INAD: Infantile Neuroaxonal Dystrophy
INAS: Inventory of Non-Ataxia Symptoms
Indel: Insertion/deletion
MAF: Minor allele frequency
MRI: Magnetic Resonance Imaging
NCL: Neuronal ceroid lipofuscinoses
NIMAD: National Institute for Medical Research Development
NPC: Niemann–Pick disease
SARA: Assessment and Rating of Ataxia
SCA: Spinocerebellar ataxia
TP-PCR: Triplet repeat primed PCR
VUS: Variant of Uncertain Significance
WES: Whole Exome Sequencing
WGS: Whole Genome Sequencing
